# Multicenter evaluation of the BIOFIRE Joint Infection Panel for the detection of bacteria, yeast, and AMR genes in synovial fluid samples

**DOI:** 10.1128/jcm.00357-23

**Published:** 2023-10-25

**Authors:** Jaime Esteban, Llanos Salar-Vidal, Bryan H. Schmitt, Amy Waggoner, Frédéric Laurent, Lelia Abad, Thomas W. Bauer, Irving Mazariegos, Joan-Miquel Balada-Llasat, Jared Horn, Donna M. Wolk, Alexa Jefferis, Mirjam Hermans, Irma Verhoofstad, Susan M. Butler-Wu, Minette Umali-Wilcox, Caitlin Murphy, Barbara Cabrera, David Craft, Benjamin von Bredow, Amy Leber, Kathy Everhart, Jennifer Dien Bard, Irvin Ibarra Flores, Judy Daly, Rebecca Barr, Kristen Holmberg, Corrin Graue, Bart Kensinger

**Affiliations:** 1 IIS-Fundacion Jimenez Diaz, CIBERINFEC-CIBER de Enfermedades Infecciosas, Madrid, Spain; 2 Indiana University School of Medicine, Indianapolis, Indiana, USA; 3 Hospices Civils de Lyon, Lyon, France; 4 Hospital for Special Surgery, New York, USA; 5 The Ohio State University Wexner Medical Center, Columbus, Ohio, USA; 6 Geisinger Health, Danville, Pennsylvania, USA; 7 Jeroen Bosch Hospital, Den Bosch, the Netherlands; 8 University of Southern California, Los Angeles, California, USA; 9 University of Nebraska Medical Center Omaha, Omaha, Nebraska, USA; 10 The Milton S. Hershey Medical Center, Hershey, Pennsylvania, USA; 11 Nationwide Children’s Hospital, Columbus, Ohio, USA; 12 Children’s Hospital Los Angeles, Los Angeles, California, USA; 13 Primary Children’s Hospital, Salt Lake City, Utah, USA; 14 bioMérieux, Inc., Salt Lake City, Utah, USA; National Institute of Allergy and Infectious Diseases, Bethesda, Maryland, USA

**Keywords:** septic arthritis, PCR, diagnosis, multiplex PCR, arthritis

## Abstract

The bioMérieux BIOFIRE Joint Infection (JI) Panel is a multiplex *in vitro* diagnostic test for the simultaneous and rapid (~1 h) detection of 39 potential pathogens and antimicrobial resistance (AMR) genes directly from synovial fluid (SF) samples. Thirty-one species or groups of microorganisms are included in the kit, as well as several AMR genes. This study, performed to evaluate the BIOFIRE JI Panel for regulatory clearance, provides data from a multicenter evaluation of 1,544 prospectively collected residual SF samples with performance compared to standard-of-care (SOC) culture for organisms or polymerase chain reaction (PCR) and sequencing for AMR genes. The BIOFIRE JI Panel demonstrated a sensitivity of 90.9% or greater for all but six organisms and a positive percent agreement (PPA) of 100% for all AMR genes. The BIOFIRE JI Panel demonstrated a specificity of 98.5% or greater for detection of all organisms and a negative percent agreement (NPA) of 95.7% or greater for all AMR genes. The BIOFIRE JI Panel provides an improvement over SOC culture, with a substantially shorter time to result for both organisms and AMR genes with excellent sensitivity/PPA and specificity/NPA, and is anticipated to provide timely and actionable diagnostic information for joint infections in a variety of clinical scenarios.

## INTRODUCTION

Bone and joint infections are a group of human diseases that include several different categories and have been well known since historic times (1). These infections classically include osteomyelitis and arthritis in their acute and chronic forms. Osteomyelitis, especially its chronic form, is a very difficult-to-treat disease, often requiring extensive surgery and prolonged antibiotic treatments to obtain patient cure (2). Septic arthritis is the most aggressive form because of the risk of sepsis secondary to the disease (3, 4). In recent decades, implant-associated infections have emerged as the most frequent osteoarticular infections (5). These infections are increasing in number due to the increasing number of patients who received many different types of implants, especially joint prostheses. The use of these prostheses has revolutionized modern medicine because they have represented an essential improvement in the life quality of the patients, allowing some of them to regain mobility and avoiding immobilization in many cases. However, this approach is not free of complications, and resulting infections often require further surgeries and prolonged antibiotic treatments (frequently with combinations of drugs) that could have consequential secondary effects (5). This increasing number of multidrug-resistant organisms further complicates antibiotic selection, so the importance of a proper etiological diagnosis is more important than ever (6).

This diagnosis of joint infections is a complex procedure that includes clinical, analytical, and microbiological data. The complexity of this process has led to the development of guidelines attempting to clarify the approach to making this diagnosis (7, 8). In all these guidelines, the importance of etiological diagnosis through microbiological testing is an essential point in the process. However, joint infection diagnosis has many problems that are not solved yet.

Rapid diagnosis of these infections still relies on the classical Gram stain of the clinical samples in most laboratories. This method, despite being well-known, easy to perform, and available to all laboratories, lacks sensitivity (9). In septic arthritis, the sensitivity of the Gram stain method is about 20%–30% (10) and is even lower in prosthetic joint infections (PJIs) (11, 12). Because of this low sensitivity, etiological diagnosis must be performed by conventional cultures, which often require a lengthy time to result (13). A minimum of 72 h is needed to finalize a negative culture result, and sometimes this time can be as long as 15 days (14). Moreover, the growth of microorganisms in culture is affected by many factors (previous antibiotic treatments, fastidious pathogens, low quality of samples, etc.), preventing an etiological identification, so empirical treatment is necessary. However, it is difficult to select antibiotics because of the increasing number of multidrug-resistant organisms (15, 16). Frequently, empirical treatment can have lower efficacy than a properly selected treatment informed by microbiological testing due to the increasing detection of antimicrobial resistance among the microorganisms that are the cause of the infections (6) and/or the possibility to use more effective antibiotics that are considered of great importance for a good outcome of these infections (17).

Trying to improve the microbiological diagnosis, molecular biology is a key tool for a better and faster diagnosis of these diseases. Many laboratory-developed molecular approaches have shown enormous potential, improving sensitivity and shortening the time for a result (18, 19), but a commercially available, fast, and accurate diagnosis is still an unmet need. Here we report a multicenter, prospective study that was submitted to the U.S. Food and Drug Administration (FDA) for *de Novo* authorization as an *in vitro* diagnostic test. The results demonstrate that the BIOFIRE JI Panel is a fast and accurate tool for the detection and identification of organisms as well as antimicrobial resistance (AMR) genes associated with these organisms for use as an aid in the diagnosis of septic arthritis and PJI.

## MATERIALS AND METHODS

### Clinical specimens

The study was conducted at 13 geographically distinct U.S. and E.U. sites (Indiana University School of Medicine, Indianapolis, IN; University of Nebraska Medical Center, Omaha, NE; Hospices Civils de Lyon, Lyon, France; Keck School of Medicine of University of Southern California, Los Angeles, CA; Jeroen-Bosch Ziekenhuis, Den Bosch, the Netherlands; Hospital Universitario Fundación Jiménez Díaz, Madrid, Spain; Hospital for Special Surgery, New York, NY; Penn State Hershey Medical and Children’s Hospital, Hershey, PA; The Ohio State University Wexner Medical Center, Columbus, OH; Geisinger Health, Danville, PA; Children’s Hospital Los Angeles, Los Angeles, CA; Nationwide Children’s Hospital, Columbus, OH; and Primary Children’s Hospital, Salt Lake City, UT) over a period of approximately 1 yr and 10 mo (May 2018–March 2020). Between May 2018 and August 2019, specimens were collected and immediately frozen for later testing. Between August 2019 and March 2020, specimens were collected and tested fresh; previously enrolled, frozen specimens were also tested during this time. Residual specimens from subjects of all ages meeting the following inclusion criteria were enrolled: specimen was neat synovial fluid (i.e., has not been diluted in any type of transport media and was not collected on a swab), specimen was left over from standard-of-care (SOC) culture testing for suspected bone or joint infection (as defined by a physician-ordered culture), specimen had been placed in a refrigerator (~4°C) as soon as possible after collection and stored for less than or equal to 7 days before enrollment, sufficient volume was available for use in the study (900 µL for subjects 18 yr of age and over or 600 µL for subjects 17 yr of age and under), and specimen was from a subject that had not previously been enrolled in the study. A waiver of the requirement for informed consent was obtained from the Institutional Review Boards (IRBs) at each study site for the use of residual synovial fluid specimens and for the collection of information from the medical records of the enrolled subjects, including clinical and demographic data. Clinical and demographic data were collected, including date of specimen collection, age range at time of collection, sex, the location of the infected joint, the presence or absence of a prosthesis, the results of the clinician-ordered SOC culture, and phenotypic antimicrobial susceptibility testing (AST) of clinical isolates.

### BIOFIRE JI Panel testing

This study was conducted with an investigational-use-only (IUO) version of the BIOFIRE JI Panel that is identical to the commercial (i.e., FDA-cleared, CE-marked) *in vitro* diagnostic (IVD) version. All specimen handling occurred in a biosafety cabinet with operators wearing appropriate personal protective equipment, preparing one specimen at a time, and cleaning between specimens, all according to the manufacturer’s instructions for use (IFU) (20). Approximately 200 µL of specimen was subject to BIOFIRE JI Panel testing. The BIOFIRE JI Panel consists of automated nucleic acid extraction, reverse transcription, nucleic acid amplification, and automated results analysis in approximately 1 h per run (i.e., per specimen). If either internal control fails, the software automatically provides a result of “Invalid” for all panel analytes. Organisms and AMR genes are reported qualitatively as “Detected” or “Not Detected.” AMR genes are only reported if one or more applicable bacteria (i.e., potential carriers of the AMR gene) are also “Detected“; if no applicable bacteria are “Detected,” the AMR gene results are reported as “N/A’ (not applicable). The BIOFIRE JI Panel simultaneously detects and identifies 31 organisms and 8 AMR genes (Tables 1 and 2).

**TABLE 1:** BIOFIRE JI Panel menu interpretations

Gram−positive bacteria		
*Anaerococcus prevotii/vaginalis*	*Parvimonas micra*	*Streptococcus* spp.
*Clostridium perfringens*	*Peptoniphilus*	*Streptococcus agalactiae*
*Cutibacterium avidum/granulosum*	*Peptostreptococcus anaerobius*	*Streptococcus pneumoniae*
*Enterococcus faecalis*	*Staphylococcus aureus*	*Streptococcus pyogenes*
*Enterococcus faecium*	*Staphylococcus lugdunensis*	
*Finegoldia magna*		

Gram−negative bacteria		
*Bacteroides fragilis*	*Kingella kingae*	*Proteus* spp.
*Citrobacter*	*Klebsiella aerogenes*	*Pseudomonas aeruginosa*
*Enterobacter cloacae* complex	*Klebsiella pneumoniae* group	*Salmonella* spp.
*Escherichia coli*	*Morganella morganii*	*Serratia marcescens*
*Haemophilus influenzae*	*Neisseria gonorrhoeae*	

Yeast		
*Candida*		
*Candida albicans*		

Antimicrobial resistance genes		
CTX−M	*mecA/C* and MREJ (MRSA)	*vanA/B*
IMP	NDM	VIM
KPC	OXA−48−like	

**TABLE 2:** Antimicrobial resistance (AMR) Genes and applicable bacteria interpretations^*a*^

Antimicrobial resistance gene(s)	Applicable bacteria	
*mecA/C* and MREJ (MRSA)	*Staphylococcus aureus*	
*vanA/B*	*Enterococcus faecalis*	*Enterococcus faecium*
OXA−48−like	*Citrobacter*	*Morganella morganii*
	*Enterobacter cloacae* complex	*Proteus* spp.
	*Escherichia coli*	*Salmonella* spp.
	*Klebsiella aerogenes*	*Serratia marcescens*
	*Klebsiella pneumoniae* group	
CTX−M	*Citrobacter*	*Morganella morganii*
IMP	*Enterobacter cloacae* complex	*Pseudomonas aeruginosa*
KPC	*Escherichia coli*	*Proteus* spp.
NDM	*Klebsiella aerogenes*	*Salmonella* spp.
VIM	*Klebsiella pneumoniae* group	*Serratia marcescens*

^*a*^ If an applicable bacteria is not detected by the BIOFIRE JI Panel the AMR gene result is reported as “N/A”.

### Comparator testing

#### Standard-of-care culture

All 13 study sites followed their own standard, validated procedures to determine SOC culture results, independent of the study (Table S1). Results were obtained from a chart review of subjects’ medical information.

#### PCR and sequencing

For AMR genes, PCR assays followed by sequencing of the same gene identified by the BIOFIRE JI Panel were used as the comparator method. One assay was used for each AMR gene. Sequencing for all assays was performed using either Sanger sequencing or targeted next-generation sequencing (NGS). For all specimens, nucleic acid was extracted from the specimens using a MagNA Pure 96 instrument. Validation testing demonstrated that most PCR comparator assays had a limit of detection (LoD) that was within fivefold sensitivity to the BIOFIRE JI Panel assay, which was considered “equivalent.” Testing was performed at BioFire in a blinded manner. Assays were designed to generate amplicons that would provide sufficient sequence information for conclusive analyte identification (between 100 and 200 bp). A sequence-confirmed positive result from Sanger sequencing was considered positive. A minimum read count of 30 for targeted NGS sequencing was considered positive.

### Results and discrepant analysis

A BIOFIRE JI Panel result was considered a true positive (TP) or true negative (TN) only when it agreed with the result from the comparator method. Discrepancy analysis ensued when results were discordant, i.e., false positive (FP) or false negative (FN) results. When sufficient specimen volume was available, discordant specimens were investigated using additional, independent PCR assays. For some discrepant results, additional testing was performed on clinical isolates (additional, independent PCR assays or Vitek2). Note that the performance data for sensitivity or positive percent agreement (PPA) and specificity or negative percent agreement (NPA) presented in this manuscript consist of unresolved data as presented in the IFU (20) for the FDA-cleared test; discrepancy investigation is provided but was not used to recalculate performance data.

### Statistical analysis

The exact binomial two-sided 95% confidence intervals (95% CI) were calculated for performance measures according to the Wilson score method (21).

## RESULTS

### Demographics

During the study period, 1,544 samples from unique subjects were enrolled and tested. Sex and age information, stratified by specimens collected from a native joint, a joint containing a prosthesis, or whether this information was unknown, is shown in Table 3. Eight hundred fifty samples were collected from native joints (i.e., joints did not contain a prosthesis), and 442 samples were from prosthetic joints (i.e., joints contained a prosthesis). For 252 subjects, whether the joint contained a prosthesis or not was unable to be extracted from the subject’s medical record. For subjects in which specimens were collected from native joints, the median age group was 26–64 yr and 61.2% were male, while the median age group was 65+ yr, and 48.2% were male for subjects in which specimens were collected from prosthetic joints.

**TABLE 3:** Sex and age demographics of subjects stratified by presence or absence of a prosthetic in the joint from which the specimen was obtained

Category		Native joint	Prosthesis	Unknown
Sex	Male	520 (61.2%)	213 (48.2%)	145 (57.5%)
	Female	330 (38.8%)	229 (51.8%)	107 (42.5%)
Age	≤4 yr	23 (2.7%)	0 (0%)	0 (0%)
	5–15 yr	70 (8.2%)	2 (0.5%)	3 (1.2%)
	16–25 yr	30 (3.5%)	2 (0.5%)	3 (1.2%)
	26–64 yr	466 (54.8%)	177 (40.0%)	131 (52.0%)
	≥65 yr	261 (30.7%)	261 (59.0%)	115 (45.6%)
Total		850	442	252

### BIOFIRE JI Panel test performance

Two hundred two specimens were positive by culture for at least one on-panel organism, and 242 specimens were positive by the BIOFIRE JI Panel for at least one organism. The overall performance of the BIOFIRE JI Panel for organisms was 90.5% sensitivity and 99.6% specificity. The complete performance for all organism results is provided in Table 4. For the FN discrepancies, 20 results were positive by culture/negative by the BIOFIRE JI Panel, and for the FP discrepancies, 70 results were negative by culture/positive by the BIOFIRE JI Panel. A total of 70 specimens grew 75 off-panel microorganisms (not included in the test), and these results were not considered as FNs. A summary of the off-panel organisms identified by SOC culture is presented in Table 5. The discrepant results were analyzed by testing the sample with additional PCR assays, confirming the detection of the microorganisms in 14 (70.0%) of the FN results, supporting the reference method as being correct. The BIOFIRE JI Panel result was confirmed for two (10%) of the FN results, and an additional four (20%) were unable to be resolved by discrepancy investigation. Additional PCR assays also confirmed the detection of the microorganisms in 76 (96.2%) of the FP results, supporting the BIOFIRE JI Panel result as being correct. An additional three (3.8%) of the FP results were unable to be resolved by a discrepancy investigation. A summary of the discrepancy investigation outcomes is presented in Table 6.

**TABLE 4:** Organism interpretations performance for the BIOFIRE JI Panel as presented in the IFU (20)

Analyte	Sensitivity			Specificity		
	TP/(TP + FN)	%	95% CI	TN/(TN + FP)	%	95% CI
Gram−positive bacteria						
*Anaerococcus prevotii/vaginalis*	1/1	100	−	1543/1543	100	99.8%–100%
*Clostridium perfringens*	0/0	−	−	1544/1544	100	99.8%–100%
*Cutibacterium avidum/granulosum*	0/0	−	−	1544/1544	100	99.8%–100%
*Enterococcus faecalis*	10/10	100	72.2%–100%	1529/1534	99.7	99.2%–99.9%
*Enterococcus faecium*	1/1	100	−	1541/1543	99.9	99.5%–100%
*Finegoldia magna*	3/3	100	43.9%–100%	1540/1541	99.9	99.6%–100%
*Parvimonas micra*	0/1	0	−	1543/1543	100	99.8%–100%
*Peptoniphilus*	1/1	100	−	1542/1543	99.9	99.6%–100%
*Peptostreptococcus anaerobius*	0/0	−	−	1541/1544	99.8	99.4%–99.9%
*Staphylococcus aureus*	98/105	93.3	86.9%–96.7%	1417/1439	98.5	97.7%–99.0%
*Staphylococcus lugdunensis*	2/2	100	34.2%–100%	1539/1542	99.8	99.4%–99.9%
*Streptococcus* spp.	38/44	86.4	73.3%–93.6%	1488/1500	99.2	98.6%–9.5%
*Streptococcus agalactiae*	10/11	90.9	62.3%–98.4%	1532/1533	99.9	99.6%–100%
*Streptococcus pneumoniae*	3/3	100	43.9%–100%	1541/1541	100	99.8%–100%
*Streptococcus pyogenes*	11/12	91.7	64.6%–98.5%	1532/1532	100	99.7%–100%
Gram−negative bacteria						
*Bacteroides fragilis*	0/0	−	−	1543/1544	99.9	99.6%–100%
*Citrobacter*	2/2	100	34.2%–100%	1542/1542	100	99.8%–100%
*Enterobacter cloacae* complex	2/4	50.0	15.0%–85.0%	1538/1540	99.9	99.5%–100%
*Escherichia coli*	14/14	100	78.5%–100%	1529/1530	99.9	99.6%–100%
*Haemophilus influenzae*	1/1	100	−	1542/1543	99.9	99.6–100%
*Kingella kingae*	1/1	100	−	1537/1543	99.6	99.2–99.8%
*Klebsiella aerogenes*	0/0	−	−	1544/1544	100	99.8–100%
*Klebsiella pneumoniae* group	4/5	80.0	37.6%–96.4%	1538/1539	99.9	99.6%−–100%
*Morganella morganii*	1/1	100	−	1541/1543	99.9	99.5%–100%
*Neisseria gonorrhoeae*	2/2	100	34.2%–100%	1539/1542	99.8	99.4%–99.9%
*Proteus* spp.	4/4	100	51.0%–100%	1536/1540	99.7	99.3%–99.9%
*Pseudomonas aeruginosa*	2/2	100	34.2%–100%	1539/1542	99.8	99.4%–99.9%
*Salmonella* spp.	0/0	−	−	1544/1544	100	99.8%–100%
*Serratia marcescens*	2/2	100	34.2%–100%	1541/1542	99.9	99.6%–100%
Yeast						
*Candida*	4/7	57.1	25.0%–84.2%	1536/1537	99.9	99.6%–100%
*Candida albicans*	3/5	60.0	23.1%–88.2%	1539/1539	100	99.8%–100%

**TABLE 5:** Off−panel organisms identified by SOC culture (*N* = 70 specimens)

Off−Panel organism identified	Number identified
*Staphylococcus epidermidis*	38
*Cutibacterium acnes*	8
*Staphylococcus capitis*	5
*Corynebacterium striatum*	3
*Staphylococcus hominis*	3
*Corynebacterium amycolatum*	2
*Staphylococcus caprae*	2
*Acinetobacter baumannii* complex	1
*Arthrobacter cumminsii*	1
*Bacillus licheniformis*	1
*Capnocytophaga canimorsus*	1
*Clostridium symbiosum*	1
*Enterococcus gallinarum*	1
*Enterococcus hirae*	1
*Klebsiella oxytoca*	1
*Granulicatella adiacens*	1
*Pasteurella multocida*	1
*Prevotella intermedia*	1
*Staphylococcus haemolyticus*	1
*Staphylococcus saccharolyticus*	1
*Staphylococcus warneri*	1
Total	75

**TABLE 6:** Summary of discrepancy investigation outcomes

Result and analyte	No. of results	No. of investigations		
		Comparator result confirmed	BIOFIRE JI result confirmed	Inconclusive
BIOFIRE JI Panel FN				
*Parvimonas micra*	1	1	0	0
*Staphylococcus aureus*	7	5	1^*a*^	1
*Streptococcus* spp.	6	4	0	2
*Streptococcus agalactiae*	1	1	0	0
*Streptococcus pyogenes*	1	0	0	1
*Enterobacter cloacae* complex	2	1	1^*b*^	0
*Klebsiella pneumoniae* group	1	1	0	0
*Candida*	3	2	0	1
*Candida albicans*	2	1	0	1
Total	20	14	2	4
% of total FN results		70.0%	10.0%	20.0%
BIOFIRE JI Panel FP				
*Enterococcus faecalis*	5	0	5	0
*Enterococcus faecium*	2	0	2	0
*Finegoldia magna*	1	0	1	0
*Peptoniphilus*	1	0	1	0
*Peptostreptococcus anaerobius*	3	0	3	0
*Staphylococcus aureus*	22	0	19	3
*Staphylococcus lugdunensis*	3	0	3	0
*Streptococcus* spp.	12	0	12	0
*Streptococcus agalactiae*	1	0	1	0
*Bacteroides fragilis*	1	0	1	0
*Enterobacter cloacae* complex	2	0	2	0
*Escherichia coli*	1	0	1	0
*Haemophilus influenzae*	1	0	1	0
*Kingella kingae*	6	0	6	0
*Klebsiella pneumoniae* group	1	0	1	0
*Morganella morganii*	2	0	2	0
*Neisseria gonorrhoeae*	3	0	3	0
*Proteus* spp.	4	0	4	0
*Pseudomonas aeruginosa*	3	0	3	0
*Serratia marcescens*	1	0	1	0
*Candida*	1	0	1	0
*mecA/C* and MREJ (MRSA)	4	0	4	0
Total	79	0	76	3
% of total FP results		0%	96.2%	3.8%

^*a*^ investigation was indicative of isolate misidentification: *S. argenteus.*

^*b*^ investigation was indicative of isolate misidentification: *E. bugandensis.*

The overall performance of the BIOFIRE JI Panel for AMR genes was 100% PPA and 98.8% NPA and is presented in Table 7. Of the four total FPs, all were for *mecA/C* and MREJ (MRSA) results, and all were confirmed to be methicillin-resistant *Staphylococcus aureus* (MRSA) by either additional PCR assays or phenotypic identification of MRSA by SOC.

**TABLE 7:** AMR gene performance for the BIOFIRE JI Panel as presented in the IFU (20)

Analyte	Positive percent agreement			Negative percent agreement		
	TP/(TP + FN)	%	95% CI	TN/(TN + FP)	%	95% CI
Antimicrobial resistance genes						
CTX−M	5/5	100	56.6%–100%	33/33	100	89.6%–100%
IMP	0/0	−	−	38/38	100	90.8%–100%
KPC	0/0	−	−	40/40	100	91.2%–100%
*mecA/C* and MREJ (MRSA)	19/19	100	83.2%–100%	90/94	95.7	89.6%–98.3%
NDM	0/0	−	−	40/40	100	91.2%–100%
OXA−48−like	1/1	100	−	33/33	100	89.6%–100%
*vanA/B*	3/3	100	43.9%–100%	14/14	100	78.5%–100%
VIM	0/0	−	−	38/38	100	90.8%–100%

Performance for the organisms was additionally stratified by specimens collected from native joints (*N* = 850) and specimens collected from prosthetic joints (*N* = 442). Some organism interpretations were combined into higher classification groups, such as Enterobacterales and gram-positive anaerobes, to increase power due to smaller sample sizes. Only on-panel organisms are discussed below. For specimens collected from native joints, sensitivity and specificity were 88.2% and 99.6%, respectively; performance with interpretations combined into functional groups for increased power is presented in Table 8. For specimens collected from prosthetic joints, overall organism sensitivity and specificity were 92.0% and 99.4%, respectively; performance with interpretations combined into functional groups for increased power is presented in Table 9.

**TABLE 8:** Grouped organism performance for specimens obtained from native joints (i.e., no prosthesis)

No prosthesis (*N* = 850)	Sensitivity			Specificity		
Analyte	TP/(TP + FN)	%	95% CI	TN/(TN + FP)	%	95% CI
gram−positive anaerobes^*a*^	0/0	−	−	848/850	99.8	99.1%–99.9%
*Enterococcus* spp.^*b*^	4/4	100	51.0%–100%	842/846	99.5	98.8%–99.8%
*Staphylococcus aureus*	45/50	90.0	78.6%–95.7%	789/800	98.6	97.6%–99.2%
*Staphylococcus lugdunensis*	2/2	100	34.2%–100%	847/848	99.9	99.3%–100%
*Streptococcus* spp.^*c*^	16/19	84.2	62.4%–94.5%	826/831	99.4	98.6%–99.7%
*Bacteroides fragilis*	0/0	−	−	849/850	99.9	99.3%–100%
Enterobacterales^*d*^	10/12	83.3	55.2%–95.3%	835/838	99.6	99.0%–99.9%
*Haemophilus influenzae*	1/1	100	−	849/849	100	99.5%–100%
*Kingella kingae*	1/1	100	−	844/849	99.4	98.6%–99.7%
*Neisseria gonorrhoeae*	2/2	100	34.2%–100%	845/848	99.6	99.0%–99.9%
*Pseudomonas aeruginosa*	0/0	−	−	849/850	99.9	99.3%–100%
Candida	1/2	−	−	848/848	100	99.5–100%
Candida albicans				848/848	100	99.5–100%

AMR gene	Positive percent agreement			Negative percent agreement		
	TP/(TP + FN)	%	95% CI	TN/(TN + FP)	%	95% CI
CTX−M	2/2	100	34.2%–100%	11/11	100	74.1%–100%
IMP	0/0	−	−	13/13	100	77.2%–100%
KPC	0/0	−	−	14/14	100	78.5%–100%
NDM	0/0	−	−	14/14	100	78.5%–100%
OXA−48−like	0/0	−	−	13/13	100	77.2%–100%
VIM	0/0	−	−	13/13	100	77.2%–100%
*mecA/C* and MREJ (MRSA)	9/9	100	70.1%–100%	44/46	95.7	85.5%–98.8%
*vanA/B*	3/3	100	43.9%–100%	4/4	100	51.0%–100%

^*a*^ *Anaerococcus prevotii/vaginalis*, *Clostridium perfringens*, *Cutibacterium avidum/granulosum*, *Finegoldia magna*, *Parvimonas micra*, *Peptoniphilus*, and *Peptostreptococcus anaerobius* interpretations have been combined and overall performance is shown.

^*b*^ *Enterococcus faecalis* and *Enterococcus faecium* interpretations have been combined and overall performance is shown.

^*c*^ *Streptococcus agalactiae*, *Streptococcus pneumoniae*, and *Streptococcus pyogenes* interpretations have been omitted from this analysis and only the redundant *Streptococcus* spp. interpretation’s performance is shown.

^*d*^ *Citrobacter*, *Enterobacter cloacae* complex, *Escherichia coli*, *Klebsiella aerogenes*, *Klebsiella pneumoniae* group, *Morganella morganii*, *Proteus* spp., *Salmonella* spp., *and Serratia marcescens* interpretations have been combined and overall performance is shown.

**TABLE 9:** Grouped organism performance for specimens obtained from joints containing a prosthesis

Prosthesis (*N* = 442)	Sensitivity			Specificity		
Analyte	TP/(TP + FN)	%	95% CI	TN/(TN + FP)	%	95% CI
gram−positive anaerobes^*a*^	3/4	75.0	30.1%–95.4%	437/438	99.8	98.7%–100%
*Enterococcus* spp.^*b*^	5/5	100	56.6%–100%	434/437	99.3	98.0%–99.8%
*Staphylococcus aureus*	45/46	97.8	88.7%–99.6%	385/396	97.2	95.1%–98.4%
*Staphylococcus lugdunensis*	0/0	−	−	440/442	99.5	98.4%–99.9%
*Streptococcus* spp.^*c*^	19/22	86.4	66.7%–95.3%	414/420	98.6	96.9%–99.3%
*Bacteroides fragilis*	0/0	−	−	442/442	100	99.1%–100%
Enterobacterales^*d*^	15/16	93.8	71.7%–98.9%	422/426	99.1	97.6%–99.6%
*Haemophilus influenzae*	0/0	−	−	442/442	100	99.1%–100%
*Kingella kingae*	0/0	−	−	442/442	100	99.1%–100%
*Neisseria gonorrhoeae*	0/0	−	−	442/442	100	99.1%–100%
*Pseudomonas aeruginosa*	2/2	100	34.2%–100%	438/440	99.5	98.4%–99.9%
*Candida*	3/5	60.0	23.1%–88.2%	436/437	99.8	98.7%–100%
Candida albicans	2/3	66.7	20.8-93.9%	439/439	100	99.1–100%

AMR gene	Positive percent agreement			Negative percent agreement		
	TP/(TP + FN)	%	95% CI	TN/(TN + FP)	%	95% CI
CTX−M	3/3	100	43.9%–100%	19/19	100	83.2%–100%
IMP	0/0	−	−	22/22	100	85.1%–100%
KPC	0/0	−	−	23/23	100	85.7%–100%
NDM	0/0	−	−	23/23	100	85.7%–100%
OXA−48−like	1/1	100	−	17/17	100	81.6%–100%
VIM	0/0	−	−	22/22	100	85.1%–100%
*mecA/C* and MREJ (MRSA)	10/10	100	72.2%–100%	38/40	95.0	83.5%–98.6%
*vanA/B*	0/0	−	−	8/8	100	67.6%–100%

^*a*^ *Anaerococcus prevotii/vaginalis*, *Clostridium perfringens*, *Cutibacterium avidum/granulosum*, *Finegoldia magna*, *Parvimonas micra*, *Peptoniphilus*, and *Peptostreptococcus anaerobius* interpretations have been combined and overall performance is shown.

^*b*^ *Enterococcus faecalis* and *Enterococcus faecium* interpretations have been combined and overall performance is shown.

^*c*^ *Streptococcus agalactiae*, *Streptococcus pneumoniae*, and *Streptococcus pyogenes* interpretations have been omitted from this analysis and only the redundant *Streptococcus* spp. interpretation’s performance is shown.

^*d*^ *Citrobacter*, *Enterobacter cloacae* complex, *Escherichia coli*, *Klebsiella aerogenes*, *Klebsiella pneumoniae* group, *Morganella morganii*, *Proteus* spp., *Salmonella* spp., *and Serratia marcescens* interpretations have been combined and overall performance is shown.

## DISCUSSION

The diagnosis of joint infection still presents difficulties despite advances in conventional culture-based techniques (sonication, prolonged incubation, use of enriched blood culture media) (5). However, even if these techniques give a positive result, the time to obtain it is still lengthy (a minimum of 1 day in almost all cases) because of the need for a positive culture (5, 8, 22, 23). In chronic infections, a delay of some days would not typically have a large impact on the management of the patient or the outcome. However, the rapid development of clinical complications in acute infections requires a different approach. A rapid and accurate diagnosis is essential for the optimal selection of the antibiotic treatment and has a high impact on the outcome of the patient, especially as multidrug resistance is a universal problem and proper antibiotic selection is more and more difficult (24). Moreover, particularly in cases of acute septic arthritis, it’s not uncommon to observe complications such as sepsis (25 – 27). In short, a rapid diagnosis can impact the morbidity and mortality of the patient. Currently, this level of speed can only be obtained by using nucleic acid amplification techniques, of which very few tests are commercially available (19, 28).

Molecular methods for joint infection *in vitro* diagnostics have been slow to develop compared to molecular *in vitro* diagnostics for other syndromes. Laboratory-developed techniques are usually based on broad-range PCR (16S rRNA), and despite the high sensitivity and specificity of this method, they need advanced molecular biology laboratories and experienced personnel; these limitations make these methods available only to specialized laboratories (18). However, in a recent survey in European countries, these techniques were the most frequently used among molecular biology-based methods for microbiological diagnosis (29). Other approaches have been based on multiplex-PCR assays, some of them commercial methods designed for the identification of microorganisms from positive blood culture bottles that were validated for their off-label use with bone and joint samples (19). The last group on the market is formed by commercial multiplex PCR designed specifically for use with bone and joint samples. However, the most widely used commercially available test in this group, the Unyvero I60ITI, has a low sensitivity (between 60% and 70%) (19), and the time for a result is approximately 4–5 h. Despite the Unyvero I60ITI being specifically designed for bone and joint infections, its low sensitivity limits its usefulness only to positive results. Moreover, some organisms of importance in specific settings (like *Kingella kingae* for pediatric patients) are not present among the Unyvero I60ITI-detected microorganisms.

In a recent study by Azad et al., the BIOFIRE JI Panel was compared against targeted metagenomic sequencing using 60 synovial fluid samples from patients with knee arthroplasty failure (44 of them diagnosed of PJI) (30). In Azad et al., the performance of the test for the detection of microorganisms included in the panel was high (91% sensitivity, with 100% specificity) but is less accurate for the diagnosis with infection because a high number of microorganisms are not included in the kit, especially *S. epidermidis* or *C. acnes*. Unfortunately, no data about the type of infection (acute vs chronic) were included in the article (30); however, because *S. epidermidis* and *C. acnes* are more prevalent in chronic infections, most of the isolates in this study were likely from chronic infections. For this reason, relying solely on the results of a test that did not include *S. epidermidis* for patients with chronic infections is not recommended. In another study by Saeed et al., samples were from patients with native septic arthritis and acute PJI (31). In Saeed et al., the results for acute PJI (107 samples) were as good as those of native septic arthritis, with a 95.3% agreement for those patients. Again, no clinical data were available in this study that allows us to know the actual agreement with the diagnosis of PJI (31).

Causative microorganisms most often implicated in cases of acute septic arthritis are relatively different from those of acute PJIs (4, 26). In septic arthritis, the most important pathogens are *S. aureus*, Enterobacterales, and other gram-negative bacilli. However, the etiological agents also include beta-hemolytic streptococci, *Neisseria gonorrhoeae* (32, 33), and *Kingella kingae* (among children) (34, 35). Again, the importance of rapid identification of the pathogen in this syndrome is high and, in some cases (septic patients), decisive. The usual rapid method for diagnosis is the Gram stain, but it lacks sensitivity (under 30% in most studies) and specificity (only morphological identification is achieved). Moreover, some pathogens (e.g. *N. gonorrhoeae* and *K. kingae*) are difficult to isolate from clinical samples, requiring selective media and/or prolonged incubation when suspected (33, 35). The BIOFIRE JI Panel includes these (and other) organisms that are common causes of acute joint infections and some other less common but clinically relevant microorganisms, like *Candida* spp. In this study, some cases with BIOFIRE JI Panel detection for these fastidious organisms are described, and it is highly probable that cases of positive PCR and negative cultures will appear when this system is used in clinical practice. The high sensitivity is accompanied by a high specificity, so these PCR positive-culture negatives are likely true positives (based on this study’s discrepancy investigation, in which 96.2% of FP were confirmed to contain the analyte of interest). Because the culture-based method, despite being considered the gold standard, has relatively limited sensitivity, many of the negative results among clinically and analytically diagnosed patients with septic arthritis are actually false negatives of the cultures.

Regarding undetected organisms (Table 5), 53 out of 75 were coagulase-negative staphylococci, and 14 were gram-positive rods (including eight *C. acnes*). While we do not have clinical data about these cases, these are common contaminants with doubtful significance in primary acute septic arthritis (but they are common pathogens in chronic implant-associated infections), so their role in these cases is a doubtful one. Only seven cases are organisms that can be considered as clinically relevant, but the low frequency made it difficult to be considered for being included in a multiplex-PCR panel with limited space. Anyway, there is always a possibility for detection of extremely uncommon microorganisms, so conventional culture must always be used together with molecular tools, as recommended elsewhere (36).

The rapid turnaround time of the BIOFIRE JI Panel is another important property of this method. Because the results can be available in about an hour, this method could possibly be used to manage patients in the emergency room or even intraoperatively in patients with acute PJI.

The main limitation of the study is the lack of clinical data on all the patients. This means that an appropriate evaluation of the results according to the clinical syndromes is an issue that needs further research. The lack of these data limits our results to a study about the specificity and sensitivity of the method when compared with microbiological results. The inclusion of clinical data (including previous antibiotic intake) could have made the evaluation more detailed regarding the diagnosis of different clinical syndromes. The significant number of rare analytes (organisms and AMR genes) for which the prospective positivity was low is another limitation. These analytes were further evaluated in archived and contrived studies (see IFU) (20). Another limitation is the fact that some organisms (especially coagulase-negative staphylococci and *Cutibacterium acnes*) are not included in the kit. Note that only eight *C. acnes* but a relatively high number of CONS were observed by SOC culture in this study. There are major challenges for molecular diagnostics in achieving both high sensitivity and high specificity for these analytes, which exist as commensal skin flora. Multiple cultures from the same patient are typically performed for standard microbiological testing, and algorithms are implemented where multiple, repeated isolations of the same organism may be required to confirm clinical relevance (37 – 39). The primary challenge is that sample contamination during the collection and handling of clinical specimens can present higher concentrations of organisms than in noncontaminated clinical specimens.

It has been said that an important limitation of commercial molecular kits is their relatively high cost (compared to culture methods). However, the cost of an acute PJI is usually higher than $50,000 USD (40), and a rapid diagnosis means a rapid administration of the treatment and likely shorter periods of hospitalization, so a lower cost of overall treatment is expected. Despite the current lack of specific cost-effectiveness studies, it is expected that molecular methods are not only useful for a rapid diagnosis but also a cost-effective approach for the diagnosis of acute osteoarticular infections. In fact, in the study by Balada-Llasat et al., it is reported that the delay in the introduction of appropriate treatment means an increased number of days with antibiotic therapy, an increased length of stay, and increased hospital costs (41). Even with the previously commented limitation for chronic infection and the manufacturer’s intended use recommending the product be used in conjunction with culture, the BIOFIRE JI Panel is a rapid, feasible, and easy technique that can be used to aid in the diagnosis of acute joint infections in almost all laboratories, even without specific molecular biology facilities.

## Data Availability

All the deidentified data used for analyses presented in this manuscript are available in supplementary material (Supplementary File). The primers used in BIOFIRE JI Panel assays and discrepancy assays are proprietary.

## SUPPLEMENTAL MATERIAL

Supplementary Table 1 jcm.00357-23-s0001.xlsx Anonymized data from all the cases used in the study.

Supplementary Table 2 jcm.00357-23-s0002.docx Table 2 of suppl. material.

ASM does not own the copyrights to Supplemental Material that may be linked to, or accessed through, an article. The authors have granted ASM a non-exclusive, world-wide license to publish the Supplemental Material files. Please contact the corresponding author directly for reuse.
