# Supplementary material for: Multicenter evaluation of the BIOFIRE Joint Infection Panel for the detection of bacteria, yeast, and AMR genes in synovial fluid samples
Source: J Clin Microbiol. 2023 Oct 25;61(11):e00357-23. doi: 10.1128/jcm.00357-23 (PMC10662359; doi:10.1128/jcm.00357-23)
Supplement: Supplementary Table 2 — Table 2 of suppl. material. [file jcm.00357-23-s0002.docx]

**Supplementary Materials:**

Supplementary Table 2: Summary of Each Clinical Site's Standard Operating Procedure for SF Culture^a^

| **Site Number** | **Aerobic Plates**  **(Hold Time)** | **Anaerobic Plates**  **(Hold Time)** | **Enrichment Broths**  **(Hold Time)** | **Blood Culture Bottles**  **(Hold Time)** |
| --- | --- | --- | --- | --- |
| **1** | BAP (3d)  CHOC, MAC, CNA (2d) | ABAP, PEA (2d) | THIO (5d) | - |
| **2** | CHOC (5d^b^) | ABAP (3d) | THIO (5d^b^) | BPP (5d) |
| **3** | BAP (2d)  CHOC (2d^b^) | SCS (5d^b^) | SB (14d) | - |
| **4**^c^ | BAP, CHOC, MAC (7d) | BRUHK^d^ (7d) | - | BP, BL (5d^b^) |
| **5** | BAP, CHOC, MAC, CNA (3d)  MTM^d^ (3d) | SCS (5d) | THIO (5d) | BL (5d) |
| **6** | BAP, CHOC (2d)  MAC (1d) | SCS (5d) | - | - |
| **7** | BAP, CHOC, MAC, CNA (5d) | ABAP (5d^b^)  KV (14d^b^) | THIO (5d) | - |
| **8** | BAP, CHOC, MAC (3d) | - | CMG (14d) | - |
| **9** | BAP, CHOC, MAC (2d) | SCS, PEA, LKV (6d^b^) | - | - |
| **10** | BAP (5d)  CHOC ^d^ (5d) | - | - | BP, BL (14d) |
| **11** | BAP, CHOC, MAC (2d)  CNA ^d^ (2d) | - | - | BP, BL (5d) |
| **12** | BAP, CHOC, MAC, MTM (4d) | - | THIO (4d) | - |
| **13** | BAP, CHOC, MAC, CNA (7d) | - | THIO (7d) | BP, BL (7d) |

^a^ BAP=Blood Agar; CHOC=Chocolate Agar; MAC=MacConkey Agar; CNA=Columbia Colistin Nalidixic Acid Agar; MTM=Thayer Martin Modified Agar; ABAP=CDC Anaerobic Blood Agar; SCS=Schaedler Blood Agar; PEA=Phenylalcohol Blood Agar; KV=Blood Agar + Kanamycin + Vancomycin; LKV=Brucella Laked Blood Agar + Kanamycin + Vancomycin; BRUHK=Brucella Blood Agar + Hemin + Vitamin K; CMG=Chopped Meat Glucose Broth; SB=Schaedler Broth; THIO=Thioglycollate Broth; BP=BACTEC Plus Aerobic Medium; BL=BACTEC Lytic Anaerobic Medium; BPP=BACTEC Peds Plus Medium

^b^ Hold time may be longer depending on special request from clinician

^c^ Culture SOP was amended in October of 2018 and the inoculation of BACTEC Plus and BACTEC Lytic blood culture bottles replaced enrichment with Thioglycollate broth. Before this date no blood culture bottles were inoculated and the specimen was enriched with Thioglycollate under aerobic and anaerobic conditions

^d^ Conditional plate depending on Gram stain results, presence of a prosthesis, expected contamination of other plates, or fungal culture specifically ordered
